# How does *Agrobacterium* virulence protein VirE2 hijack host actomyosin motility system?

**DOI:** 10.1080/19420889.2017.1415599

**Published:** 2017-12-26

**Authors:** Qinghua Yang, Haitao Tu, Shen Q. Pan

**Affiliations:** a Department of Biological Sciences, National University of Singapore, Singapore; b Foshan Institute of Molecular Bio-Engineering, School of Stomatology and Medicine, Foshan University, Foshan, China

**Keywords:** VirE2, protein trafficking, myosin, endoplasmic reticulum, actin filaments

## Abstract

*Agrobacterium tumefaciens* has the ability to transform a wide range of eukaryotes by transferring single-stranded (ss) DNA (T-DNA) into the host cells. During the process, the bacterium also delivers abundant amount of ssDNA binding protein VirE2 into the host cytoplasm, where multiple VirE2 molecules bind and coat the T-DNA and thus generate large-sized nucleoprotein complex. VirE2 trafficking should be representative of T-DNA movement. Recently, we demonstrated that plant actomyosin motility system facilitated the trafficking of *Agrobacterium*-delivered VirE2 inside host cytoplasm. We showed that actomyosin-driven VirE2 moved towards the nucleus along the endoplasmic reticulum (ER). Here we hypothesize that the presence of VirE2 on the cytoplasmic side of the ER may provide VirE2 with convenient access to the opening of nuclear pore complexes (NPCs). We propose models to explain how VirE2 is trafficked in the context of actin, myosin and ER.

*A. tumefaciens* infects plant cells in a series of events. First, it senses phenolic compounds secreted by wounded plant tissues. Then the virulence (*vir*) genes encoded on the tumor-inducing (Ti) plasmid is activated through a VirA-VirG two-component system.^1,2^ Subsequently, one of the *vir* gene products VirD2 generates ssT-DNA strand by nicking the T-border sequences of the Ti- plasmid and remains covalently bound to the T-DNA.^3,4^ The VirD2-T-DNA complex is then delivered together with other virulence proteins into host cells.^5^ Inside plant cells, the VirD2-bound T-DNA is coated by ssDNA binding protein VirE2, which results in the formation of nucleoprotein complex (T-complex)^6,7^ As a large size complex, the T-complex is unlikely able to move effectively through the dense cytoplasm by Brownian diffusion.^8^ Its intracellular movement should rely on an active transport system inside the cytoplasm. The mode of this active transport has been a subject of considerable interest.^9^

Recently, we demonstrated that VirE2 is trafficked inside plant cytoplasm via a myosin XI-K-powered ER/actin network.^10^ This may represent how T-complex is trafficked inside host cells, since its surface consists of VirE2 molecules. By using a split-GFP approach to visualize the bacterium-delivered VirE2, we were able to monitor the VirE2 movement in real time upon its translocation into plant cytoplasm.^11^ For the first time, VirE2 was visualized in real time to be trafficking towards the plant nucleus along a linear cellular structure that was illustrated by free DsRed molecules. This linear structure was later determined to be part of the ER/actin network, based on the experiments by chemical treatments and fluorescent marker labelling. Moreover, the GFP1–10 fragment localized inside the ER lumen did not complement the bacterium-delivered VirE2-GFP11, suggesting that the VirE2 is on the cytosolic side of the ER. Our data also showed that myosin XI-K provided the driving force for the VirE2 movement, based on the experiments by selective chemical inhibition and dominant negative interruption of myosin activity.^10^

The association of VirE2 with the ER is consistent with other studies. It was reported previously that VirE2 could form channels that might enable ssDNA to travel through artificial membranes.^12^ This in vitro study highlighted the ability of VirE2 to be associated with the membrane. Recently, Li *et al*. demonstrated that VirE2 possesses endocytic signals and could enter the plant cell via clathrin-mediated endocytosis.^13^ Moreover, a SNARE-like protein, possibly involved in vesicle budding and fusion, was also found to have a strong interaction with VirE2.^14^ These lines of evidence suggest that the endomembrane system could be relevant to VirE2 trafficking inside host cells. This is in line with our finding that VirE2 is associated with a major endomembrane component, the ER.^10^

The presence of VirE2 on the cytoplasmic side of ER is a key finding that provides insight in two aspects. First, the continuous linkage of the ER to the outer membrane of nuclear envelope allows VirE2 to reside on the same topological surface as the opening of NTCs. Second, the ER is a dynamic structure that is involved in continuous flow and movement of lipids and proteins, which enables the associated VirE2 to move inside the cytoplasm. These two aspects might be the mechanistic reason that VirE2 can readily reach the opening of NTCs for efficient nuclear import.

It is particularly intriguing to speculate how myosin XI-K plays its role in VirE2 trafficking. Myosin XI-K carries cargos with its two globular domains and moves along actin filaments using its two motor domains, but it is not clear whether VirE2 is a cargo protein for myosin XI-K. There is a possibility that myosin XI-K recognizes VirE2 with its globular domains and drives its movement. This is a straightforward model, but currently lacks experimental evidence (Fig. 1). Alternatively, the ER-associated VirE2 may be present in a vesicle form; such a vesicle may be recognized by myosin XI-K. In this model, myosin XI-K indirectly drives the VirE2 movement by carrying the VirE2-containing vesicles (Fig. 2). Recently, some myosin XI-K specific vesicle adaptors have been identified^15^, but whether they are relevant to VirE2 trafficking is to be examined in future studies. Further, VirE2 trafficking may simply be the consequence of ER motility, where VirE2 passively follows the flow of the ER because of its association with ER. VirE2 and myosin XI-K might have no direct or indirect interactions. As the ER acquires its motility by the three-way interactions among the ER/actin/myosin^16^, ER-associated VirE2 may be trafficking solely by drifting in the flux of the ER (Fig. 3).

**Figure 1.**
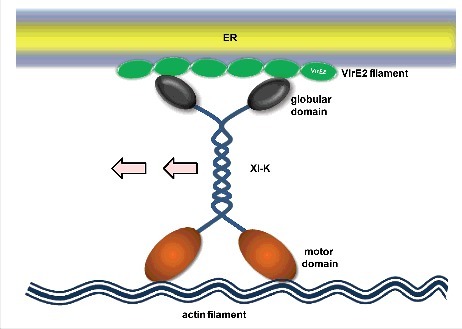
Direct cargo model. In this model, VirE2 is directly recognized and driven by myosin XI-K.

**Figure 2.**
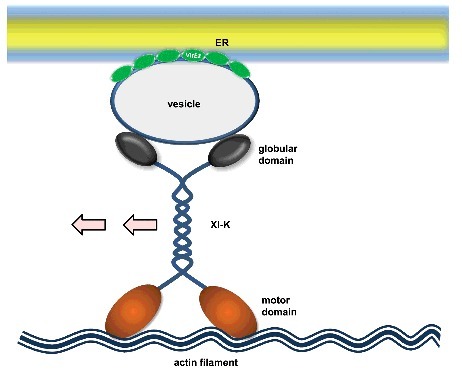
Indirect cargo model. In this model, VirE2 is not recognized by myosin XI-K; instead, it is associated with a cargo that is recognized and driven by myosin XI-K.

**Figure 3.**
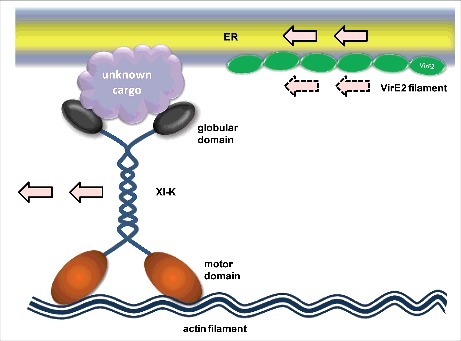
Downwind model. In this model, VirE2 is neither recognized by myosin XI-K, nor associated with a cargo that is recognized and driven by myosin XI-K; instead, it simply moves along the ER driven by myosin XI-K.
